# Do viruses require the cytoskeleton?

**DOI:** 10.1186/1743-422X-10-121

**Published:** 2013-04-18

**Authors:** Jason D Matthews, Rachel Morgan, Christie Sleigher, Teryl K Frey

**Affiliations:** 1 Department of Biology, Georgia State University, Atlanta, GA, USA

**Keywords:** Virus replication, Cytoskeleton, Microtubules, Actin filaments

## Abstract

**Background:**

It is generally thought that viruses require the cytoskeleton during their replication cycle. However, recent experiments in our laboratory with rubella virus, a member of the family Togaviridae (genus rubivirus), revealed that replication proceeded in the presence of drugs that inhibit microtubules. This study was done to expand on this observation.

**Findings:**

The replication of three diverse viruses, Sindbis virus (SINV; family Togaviridae family), vesicular stomatitis virus (VSV; family Rhabdoviridae), and Herpes simplex virus (family Herpesviridae), was quantified by the titer (plaque forming units/ml; pfu/ml) produced in cells treated with one of three anti-microtubule drugs (colchicine, noscapine, or paclitaxel) or the anti-actin filament drug, cytochalasin D. None of these drugs affected the replication these viruses. Specific steps in the SINV infection cycle were examined during drug treatment to determine if alterations in specific steps in the virus replication cycle in the absence of a functional cytoskeletal system could be detected, i.e. redistribution of viral proteins and replication complexes or increases/decreases in their abundance. These investigations revealed that the observable impacts were a colchicine-mediated fragmentation of the Golgi apparatus and concomitant intracellular redistribution of the virion structural proteins, along with a reduction in viral genome and sub-genome RNA levels, but not double-stranded RNA or protein levels.

**Conclusions:**

The failure of poisons affecting the cytoskeleton to inhibit the replication of a diverse set of viruses strongly suggests that viruses do not require a functional cytoskeletal system for replication, either because they do not utilize it or are able to utilize alternate pathways when it is not available.

## Findings

**Figure 1:**
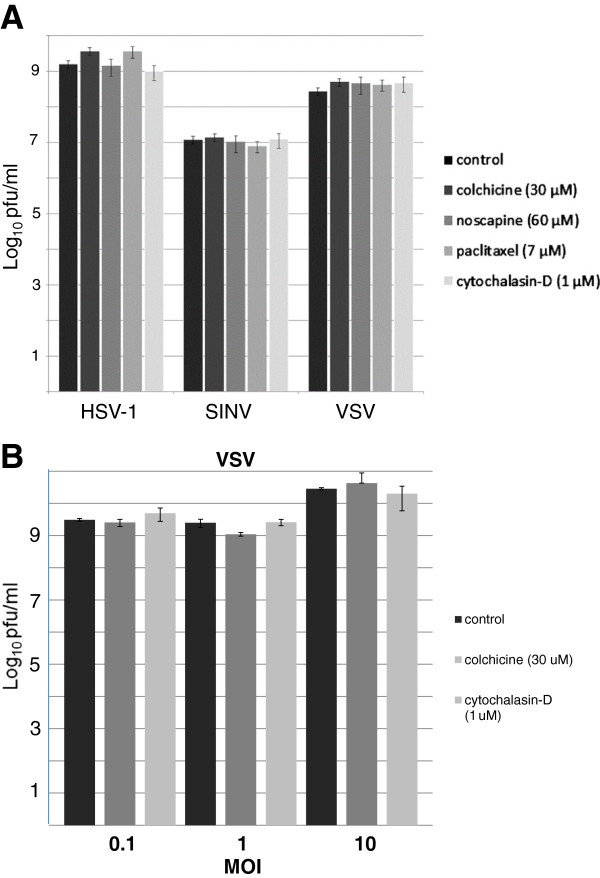
**Effect of cytoskeletal drug treatments on virus replication. A**). BHK cells were infected for 1 hour at 35°C with either Herpes Simplex virus-1 (HSV-1; multiplicity of infection (MOI) = 0.01 plaque forming unit (pfu)/cell), Sindbis virus (SINV; MOI = 0.1 pfu/cell) or vesicular stomatitis virus (VSV; MOI = 0.1 pfu/cell) and then incubated at 35°C in medium with the indicated drug. The minimal concentrations necessary to inhibit the appropriate cytoskeletal system were used as determined either by immunofluorescence staining of drug-treated, uninfected BHK cells, using antibodies against the microtubules or by phalloidin-Alexa Fluor 568 staining which binds to actin filaments, to observe changes in cytoskeletal morphology and/or inhibition of mitosis (the effects these poisons have on cells). At 24 hours post-infection, the cell culture fluid was harvested and titered by plaque assay. Results, given in log_10_ PFU/mL, were the average of three independent experiments. Error bars represent the standard deviation from the mean. **B**). BHK cells were infected for 1 hour at 4°C with VSV at MOI’s of 0.1, 1, or 10 pfu/cell. Subsequently, the cells were incubated at 35°C in medium with the indicated drug. At 24 hours post-infection, the cell culture fluid was harvested and titered by plaque assay. The results were the average of two independent experiments. Error bars represent the standard deviation from the mean.

There are three major components to the cytoskeleton; actin filaments [1], intermediate filaments [2], and microtubules [3], which *in toto* are necessary for maintenance of cell shape, cell motility and intracellular transport. It is generally thought that viruses require the cytoskeleton during infection [4], although a review of the literature reveals that most studies analyze the requirement of the cytoskeleton for specific steps in the viral replication cycle rather than the complete replication cycle. Recently, in such a study on the effects of anti-microtubule drugs on the formation of cytoplasmic fibers by a replicase protein of rubella virus, to our surprise we found that these drugs did not significantly affect the titer of virus produced [5]. To see if this finding held for other viruses, we tested the replication of three diverse viruses (Table 1) against the same panel of anti-microtubule drugs (Table 2) and also included the anti-actin filament drug, cytochalasin D. BHK (baby hamster kidney) cells (ATCC) were treated with different cytoskeletal drugs one hour after the cells were infected, and the drugs remained on the cells for the 24 hour time course of the experiment. Infection was done at a low multiplicity of infection (MOI; 0.1 pfu/cell for SINV and VSV, 0.01 pfu/cell for HSV) to ensure that multiple rounds of infection occurred, thus subjecting every step in the virus replication cycle to the presence of the drugs. Each of these viruses replicates rapidly ensuring that replication was complete during the time course of the experiment. Media harvested from untreated control or drug-treated infected BHK cells at 24 hours post-infection was titered by plaque assay to determine viral yields. None of the viruses tested exhibited a reduction in yield in cells treated with any of the anti-cytoskeleton drugs (Figure 1A), indicating that these viruses do not need a functioning cytoskeletal system to complete their replication cycle. The replication of VSV was tested at additional MOI’s (10 and 1 pfu/cell) with the same result (Figure 1B). We also compared the replication curves of VSV during drug treatments to the curves of untreated controls, all of which were infected at an MOI of 0.1 pfu/cell with a time-course of virus yield at 6, 12 and 24 hours post-infection. There were no differences in the growth kinetics for VSV between untreated or treated cultures during the time-course (data not shown).

**Table 1:** Viruses used in this study

**Virus**	**Genome**	**Family**	**Genus**	**Host**	**Site of replication**
**Herpes simplex virus (HSV-1)**	dsDNA	Herpesviridae	Simplexvirus	Human	Nucleus
**Sindbis virus (SINV)**	(+)ssRNA	Togaviridae	Alphavirus	Vertebrates; Mosquitoes	Cytoplasm
**Vesicular stomatitis virus (VSV)**	(−)ssRNA	Rhabdoviridae	Vesiculovirus	Vertebrates; Arthropods	Cytoplasm

**Table 2:** Drugs used in this study

**Drug**	**Source**	**Mode of action**	**Clinical use**
**Colchicine**	*Colchicum autmnale*	Depolymerizes microtubules	Gout treatment
**Noscapine**	Plants of the Papaveraceae family	Inhibits microtubule dynamics	Cough suppressant
**Paclitaxol**	*Taxus brevifolia*	Inhibits mitosis by stabilizing microtubules	Anti-cancer therapy
**Cytochalasin D**	*Zygosporium mansonii*	Depolymerizes actin filaments	None

**Figure 2:**
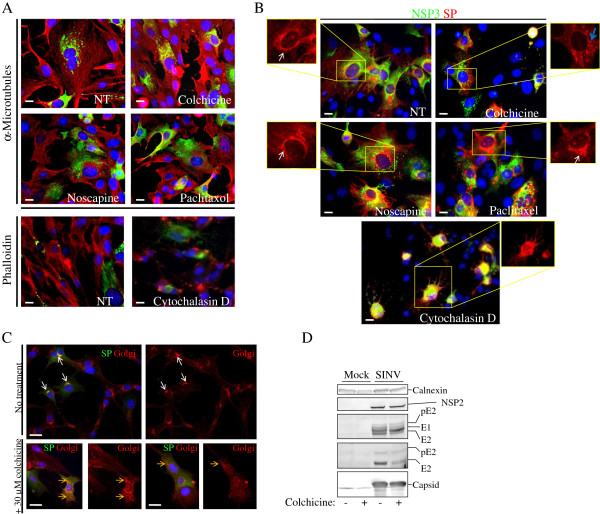
**Analysis of SINV replication during cytoskeletal drug treatment. A** and **B**. BHK cells infected at an MOI = 1 for 1 hour with SINV expressing an nsP3-GFP fusion protein (green), called SINV/NSP3-GFP, before treating the infected cells with the appropriate cytoskeletal drug for 5 hours. At 6 hours post-infection, the infected cells were stained for fluorescence microscopy with rabbit anti-microtubule antibodies (secondary antibody was donkey anti-rabbit AlexaFluor 595), the F-actin filaments with phalloidin AlexaFluor 568-conjugate (**A**), or staining for the structural proteins with rabbit antibodies raised against purified virus (visualized by donkey anti-rabbit Alexa Fluor 595-conjugate) (**B**). Insets in B (red), show localization of structural proteins. NT, no treatment. Nuclei were stained with Hoechst 33342 (blue). Bars represent 10 μm. White arrows point to juxtanuclear foci of structural proteins, blue arrow points to scattered structural protein foci. **C**. BHK cells infected with SINV (MOI = 1 pfu/cell) for 1 hour and then incubated with medium or medium containing 30 μM colchicine for an additional 5 hours were treated with a Golgi stain (wheat germ agglutinin Alexa Fluor 595 conjugate, red) along with antibodies against SINV structural proteins that were visualized with anti-rabbit FITC conjugate (green) to localize the structural proteins. Nuclei were stained with Hoechst 33342 (blue). Bars represent 20 μm. White arrows point to structural protein overlap with the Golgi. Yellow arrows point to fragmented, but overlapping, Golgi and structural proteins. **D**. Western blotting of SINV-infected (MOI = 1 pfu/cell) BHK cell lysates prepared as in **A**. Calnexin serves as a loading control. NSP2 was detected with rabbit anti-NSP2 antibody. The structural proteins, E1, pE2 and E2 were detected with rabbit polyclonal antibodies. pE2 and E2 proteins were detected with cdE2 antibodies. Protein bands were visualized with the species-specific alkaline phosphatase-conjugated antibodies and substrate NBT/BCIP.

Given our finding that three diverse viruses replicate to similar titers in the absence or presence of anti-cytoskeletal drugs, we hypothesized that either these viruses do not need the cytoskeletal system or use alternate pathways when it is not available. Since these hypotheses could apply differentially to the steps in the virus replication cycle, we used SINV to investigate whether the anti-cytoskeleton drugs had effects on specific steps in the virus replication cycle. SINV produces four nonstructural proteins (nsP1-4) that are involved in RNA-dependent RNA synthesis occurring in membranous structures in the cytoplasm of infected cells [6-8]. Using a recombinant SINV expressing a GFP-tagged nsP3 (described in [9]), we found that without drug treatment nsP3-GFP localized in perinuclear foci distributed in the cytoplasm of SINV/NSP3-GFP-infected cells (consistent with previous reports [9]) and changed little under treatment with the cytoskeletal drugs (Figure 2A and B). SINV produces three structural proteins that comprise the virus particle, the capsid protein C and envelope glycoproteins E1 and E2 [10], which is formed by budding of the nucleocapsid containing C and the genome RNA, through the plasma membrane. The intracellular distribution of the structural proteins was examined by staining with polyclonal antibodies raised in rabbits against purified SINV. Consistent with previous reports, the structural proteins were found throughout the cytoplasm of infected cells and were particularly concentrated in the perinuclear region (Figure 2B) in what has been shown to be the Golgi apparatus [11]. Neither noscapine or paclitaxel disrupted the overall distribution of the structural proteins as a whole, however colchicine disrupted the perinuclear localization of the structural proteins into fragmented foci that appeared scattered in the cytoplasm. The cytochalasin-D disrupted the shape of the infected cells making analysis of the structural protein signal difficult to localize, however they did appear to remain concentrated in the perinuclear region.

**Figure 3:**
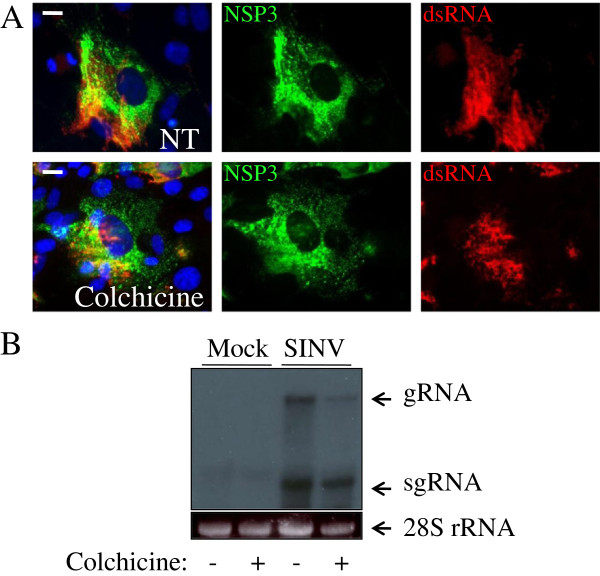
**RNA synthesis in SINV-infected cells after colchicine treatment. A**. Localization of dsRNA complexes after colchicine treatment. SINV/NSP3-GFP-infected BHK cells (MOI = 1 pfu/cell), untreated or treated with colchicine from 1–6 hours post-infection were stained at 6 hours post-infection with mouse anti-dsRNA antibodies (Scientific Consultants) and visualized with donkey anti-mouse Alexa Fluor 595 secondary antibodies (red). Nuclei are stained with Hoechst 33342 (blue) and bars represent 10 μm. **B**. Northern blotting of lysates of SINV-infected BHK cells (MOI = 1 pfu/cell) after no treatment or treatment with 30 μM colchicine (from 1–24 hours post-infection) prepared at 24 hours post-infection. SINV-specific RNAs were detected by probing with a ^32^P probe labeled by nick-translation of a plasmid containing the SINV structural protein genes.

Since colchicine has previously been shown to disrupt the Golgi [12], wheat germ agglutinin Alexa Fluor 594 conjugate was used to stain the Golgi. In untreated, SINV-infected BHK cells, the structural proteins’ signal concentrated in the perinuclear region overlapping with the Golgi signal (Figure 2C). However, the Golgi appeared fragmented or was absent in the colchicine-treated cells, but the structural protein signal still overlapped many of the fragmented Golgi foci. Western blotting of lysates from SINV-infected cells probed with anti-nsP2 or anti-structural protein antibodies revealed that viral protein synthesis was not significantly affected by colchicine, albeit with a minor decrease in levels, particularly of E2, but not of its precursor pE2 (Figure 2D). No obvious changes were observed in dsRNA distribution or abundance (a marker for sites of RNA-dependent RNA synthesis) in colchicine-treated vs untreated cells except for more staining of dsRNA around the edges of the untreated cells (Figure 3A). In contrast, Northern blotting analysis showed somewhat lower levels of SINV genome and subgenome RNA in the colchicine-treated cells than in the control cells (Figure 3B).

In summary, following studies in our lab with rubella virus [5] which found that its replication was not inhibited by four anti-microtubule drugs, we decided to test the hypothesis that viruses can replicate in the presence of drugs which compromise the cytoskeletal system by broadening our study to include another positive-strand RNA virus, a negative-strand RNA virus, and a DNA virus. Our findings demonstrate that viruses can produce normal titers in the absence of a functional cytoskeletal system, (similar results were reported for SINV in another lab [7]) which challenges the currently accepted notion [4]. In this regard, it was shown that poliovirus can complete its entire infection cycle in a cell-free system lacking a cytoskeleton system [13]. To address the alternate hypotheses of whether viruses simply do not need the cytoskeletal system or use alternate pathways when it is not available, we investigated the replication cycle of SINV in the presence of the anti-cytoskeletal drugs in more detail. No obvious changes occurred to any of the stages of SINV infection in the presence of noscapine, paclitaxel, and cytochalasin D. However, the Golgi through which the SINV envelope glycoproteins mature during transport to the plasma membrane, was severely compromised by colchicine, concomitantly affecting the distribution of the SINV structural proteins. It will be of interest to study the effect of colchicine treatment on maturation and transport of these SINV proteins to see if an alternate pathway exists that the virus uses in this step of its replication cycle in the presence of this drug.

## Competing interests

The authors declare that they have no competing interest.

## Authors’ contributions

JDM carried out the research and drafted the manuscript. RM and CS also carried out the research for their MS degrees. TKF, as senior author, advised JDM, RM and CS on the research, participated in drafting of the manuscript, and serves as corresponding author. All four authors have read and approved the final manuscript.

## Author information

RM is attending vet school at the University of Georgia. JDM is a Postdoctoral Fellow in the Department of Pathology, Emory University School of Medicine.
